# A genome-wide assessment of rare copy number variants in colorectal cancer

**DOI:** 10.18632/oncotarget.4621

**Published:** 2015-07-20

**Authors:** Zhenli Li, Dan Yu, Meifu Gan, Qiaonan Shan, Xiaoyang Yin, Shunli Tang, Shuai Zhang, Yongyong Shi, Yimin Zhu, Maode Lai, Dandan Zhang

**Affiliations:** ^1^ Department of Pathology, Zhejiang University School of Medicine, Hangzhou, Zhejiang, 310058, China; ^2^ Key Laboratory of Disease Proteomics of Zhejiang Province, Hangzhou, Zhejiang, 310058, China; ^3^ Department of Pathology, Taizhou Hospital, Linhai, Zhejiang, 317000, China; ^4^ Zhejiang University School of Clinical Medicine, Hangzhou, Zhejiang, 310058, China; ^5^ Bio-X Institutes, Key Laboratory for the Genetics of Developmental and Neuropsychiatric Disorders, Ministry of Education, Shanghai Jiao Tong University, Shanghai, 200000, China; ^6^ Department of Epidemiology & Biostatistics, Zhejiang University School of Public Health, Hangzhou, Zhejiang, 310058, China

**Keywords:** colorectal cancer, rare CNVs, genome-wide scan, nucleosome assembly

## Abstract

Colorectal cancer (CRC) is a complex disease with an estimated heritability of approximately 35%. However, known CRC-related common single nucleotide polymorphisms (SNPs) can only explain ~0.65% of the heritability. This “missing heritability” may be explained partially by rare copy number variants (CNVs). In this study, we performed a genome-wide scan using Illumina Human-Omni Express BeadChip, 694 sporadic CRC cases and 1641 controls were eventually included in our analysis after quality control. The global burden analysis revealed a 1.53-fold excess of rare CNVs in CRC cases compared with controls (*P* < 1 × 10^−6^), and the difference being more pronounced for genic rare CNVs and CNVs overlapped with coding regions (1.65-fold and 1.84-fold, respectively, both *P* < 1 × 10^−6^). Interestingly, both the cases in the lowest and middle tertile of age carried a higher burden of rare CNVs comparing to the highest tertile. Furthermore, 639 CNV-disrupted genes exclusive to CRC cases were found to be significantly enriched in gene ontology (GO) terms concerning nucleosome assembly and olfactory receptor activity. Our study was the first to evaluate the burden of rare CNVs in sporadic CRC and suggested that rare CNVs contributed to the missing heritability of CRC.

## INTRODUCTION

Colorectal cancer (CRC) is the fourth most commonly diagnosed cancer in males and the third in females worldwide [1]. In the past few decades, the incidence of CRC has increased rapidly in most Asian countries, including China [2–4].

Genetic factors are known to have crucial impacts on the incidence and development of CRC. The heritability of CRC was estimated to be approximately 35% [5]. Recent genome-wide association studies (GWAS) have identified multiple single nucleotide polymorphism (SNPs) associated with CRC [6–10]. Surprisingly, known CRC-associated SNPs can explain only ~0.65% of the heritability [11]. Copy number variants (CNVs), large deletions or duplications of DNA segments (>1kb), were considered another important form of genetic variations. It was well demonstrated that changes of copy number, which lead to aberrations of gene dosage, can influence the susceptibility to complex disease by altering gene expression [12, 13]. Plenty of studies have already highlighted the importance of CNV in cancer pathogenesis, including breast cancer, prostate cancer, neuroblastoma, etc [14–16]. Also, several CNV regions have been found to be associated with CRC. Fernandez-Rozadilla C *et al*. discovered that deletions of 11q11 were associated with increased risk of CRC [17]. In addition, several predisposing CNVs were suggested by Venkatachalam R *et al.* through GWAS on CRC [18].

However, the majority of common CNVs were in linkage disequilibrium with SNPs in the human genome, making them unlikely to account for much of the “missing heritability” for complex traits [19–21]. Increasingly, recent studies suggest that rare CNVs have substantial effects on the development of complex diseases [22, 23]. Rare CNVs have also been implicated in numerous cancers such as breast cancer, testicular cancer as well as colorectal cancer. Yang R *et al*. identified a rare deletion at 12p.12.3 in two of 384 familial CRC cases, but none in the controls, with the results being successfully validated in another independent sample [24]. Most recently, rare CNVs were displayed at protein coding genes in colorectal adenomatous polyposis [25]. Whether rare CNVs play roles in the pathogenesis of sporadic CRC cases, however, has not been examined to date. We initiated two GWASs in CRC and individuals with metabolic syndrome (MS) with shared control data set (not published). 1008 CRC cases, 998 MS and 996 controls from China were genotyped using Illumina Human-Omni Express BeadChip. In this study, we generated CNV calls from GWAS data and investigated potential contributions of rare CNVs to sporadic CRC.

## RESULTS

### Characteristics of the study population

After strict quality control, 694 CRC cases (including 336 individuals with colon cancer, 340 individuals with rectal cancer and 18 individuals with both colon and rectal cancer) and 1641 controls (the information of MS controls and non-MS controls after quality control were shown in Supplementary Table 1) were finally included in our analysis (see Table 1). Genotype results from 23 pairs of duplicate samples showed ~99.9% concordance. The first two principle components of CRC cases, non-MS controls and MS controls from PCA analysis and QQ plot for rare CNVs span a particular position were plotted in Supplementary Figure 1 and Supplementary Figure 2, respectively. None of the remained samples was removed as an outlier according to the PCA analysis. There was no statistical difference in gender between cases and controls. The age of the CRC group was significantly higher than that of the control group (*P* < 0.001).

**Table 1:** Basic characteristics of the study subjects

	CRC cases			Controls	*P* value ^a^
	All *	Colon	Rectal		
**N**	694	336	340	1641	
**Gender(M/F)**	382/312	195/141	175/165	850/791	0.151
**Age(years)**	62.4 ± 12.3	62.9 ± 12.8	62.2 ± 11.5	57.4 ± 11.6	<0.001

* As 18 of the CRC cases diagnosed with both colon and rectal cancer, they were excluded in the following stratification analysis for tumor site.

a The *P* value for gender was calculated by χ^2^ test between all the cases and controls, while the *P* value of the age between the two groups was derived from independent *T* test.

### Global burden analysis

**Figure 1:**
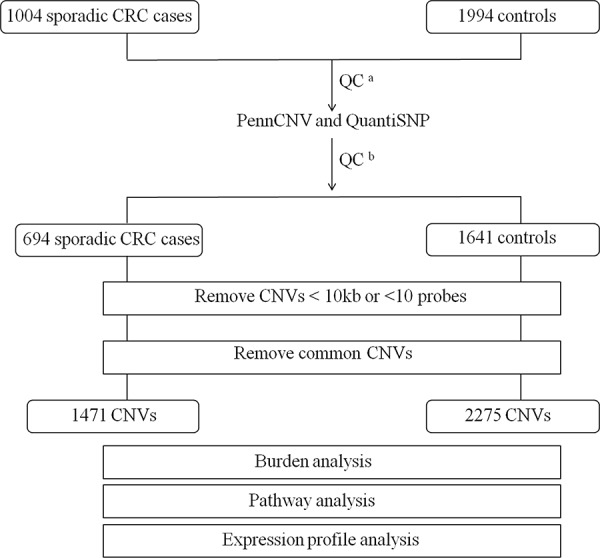
Outline of CNV discovery and CNV analysis A total of 1004 sporadic CRC cases and 1994 controls were genotyped using Illumina Human-Omni Express BeadChip. ^a^QC for SNP array data, individuals with call rate <95% or outliers were removed. ^b^QC for sample and CNV calls by PennCNV and QuantiSNP.

A total of 1471 and 2275 autosomal rare CNVs were detected for qualified cases and controls respectively (Figure 1). The number of rare CNVs per person was significantly higher in CRC cases vs. controls (2.12 vs. 1.39, *P* < 1.0 × 10^−6^, Table 2). Both deletions and duplications were enriched in CRC cases (*P* < 1.0 × 10^−6^, *P* = 2.0 × 10^−6^, respectively). The proportion of CRC cases with at least one rare deletion was significant higher than controls (0.80 vs 0.74, *P* = 0.001). No significant difference between cases and controls was found in total CNV size or average CNV size span per individual. When excluding individuals with MS, a greater burden remained in CRC patients comparing to non-MS controls (see Supplementary Table 2). There was no significant difference in the frequency of rare CNVs between males and females either in all samples or stratified into cases and controls (Supplementary Table 3).

**Table 2:** Global burden of rare CNVs between colorectal cases and controls

Category	Controls (*N* = 1641)	CRC (*N* = 694)	Fold Change^#^	*P* value*	Colon (*N* = 336)	Fold Change^#^	*P* value*	Rectal (*N* = 340)	Fold Change^#^	*P* value*
**Total number of rare CNVs**										
Total	2275	1471			751			682		
Deletion	1184	845			431					
Duplication	1091	626			320			293		
**Number of rare CNVs per sample**										
Total	1.39	2.12	1.53	**<1.0 × 10^−6^**	2.24	1.61	**<1.0 × 10^−6^**	2.01	1.45	**<1.0 × 10^−6^**
Deletion	0.72	1.22	1.69	**<1.0 × 10^−6^**	1.28	1.78	**<1.0 × 10^−6^**	1.14	1.59	**<1.0 × 10^−6^**
Duplication	0.66	0.90	1.36	**2.0 × 10^−6^**	0.95	1.43	**0.00003**	0.86	1.30	**0.0003**
**Proportion of samples with one or more rare CNVs**										
Total	0.74	0.80	1.08	**0.001**	0.80	1.09	**0.007**	0.79	1.07	**0.02**
Deletion	0.49	0.58	1.17	**0.00009**	0.59	1.19	**0.0009**	0.56	1.14	**0.01**
Duplication	0.47	0.51	1.09	**0.03**	0.49	1.04	0.30	0.54	1.15	**0.01**
**Total length of rare CNVs spanned per sample (in kb)**										
Total	234.70	258.40	1.10	0.53	251.40	1.07	0.23	265.50	1.13	0.10
Deletion	139.70	157.30	1.13	0.57	171.90	1.23	0.11	141.20	1.01	0.41
Duplication	221.90	224.50	1.01	0.58	206.40	0.93	0.74	243.10	1.10	0.19

* Empirical *p*-values between cases and controls were calculated using 1000,000 permutations by PLINK, and all the *P* values were shown in bold if reached statistical significance (*P* < 0.05).

# Fold change of CRC/colon/rectal cases vs controls.

We further stratified CRC cases into colon and rectal cancer and derived similar results. The number of rare CNVs per person was significantly enriched in colon cases/rectal cases vs. controls (1.61-fold and 1.45-fold, respectively). The proportions of samples with one or more rare deletions were significantly higher in both colon cancer patients and rectal patients than in controls (*P* = 0.0009 and *P* = 0.01, respectively). A significant increase in proportion of rectal cancer but not colon cancer with at least one duplication was observed (*P* = 0.01).

**Figure 2:**
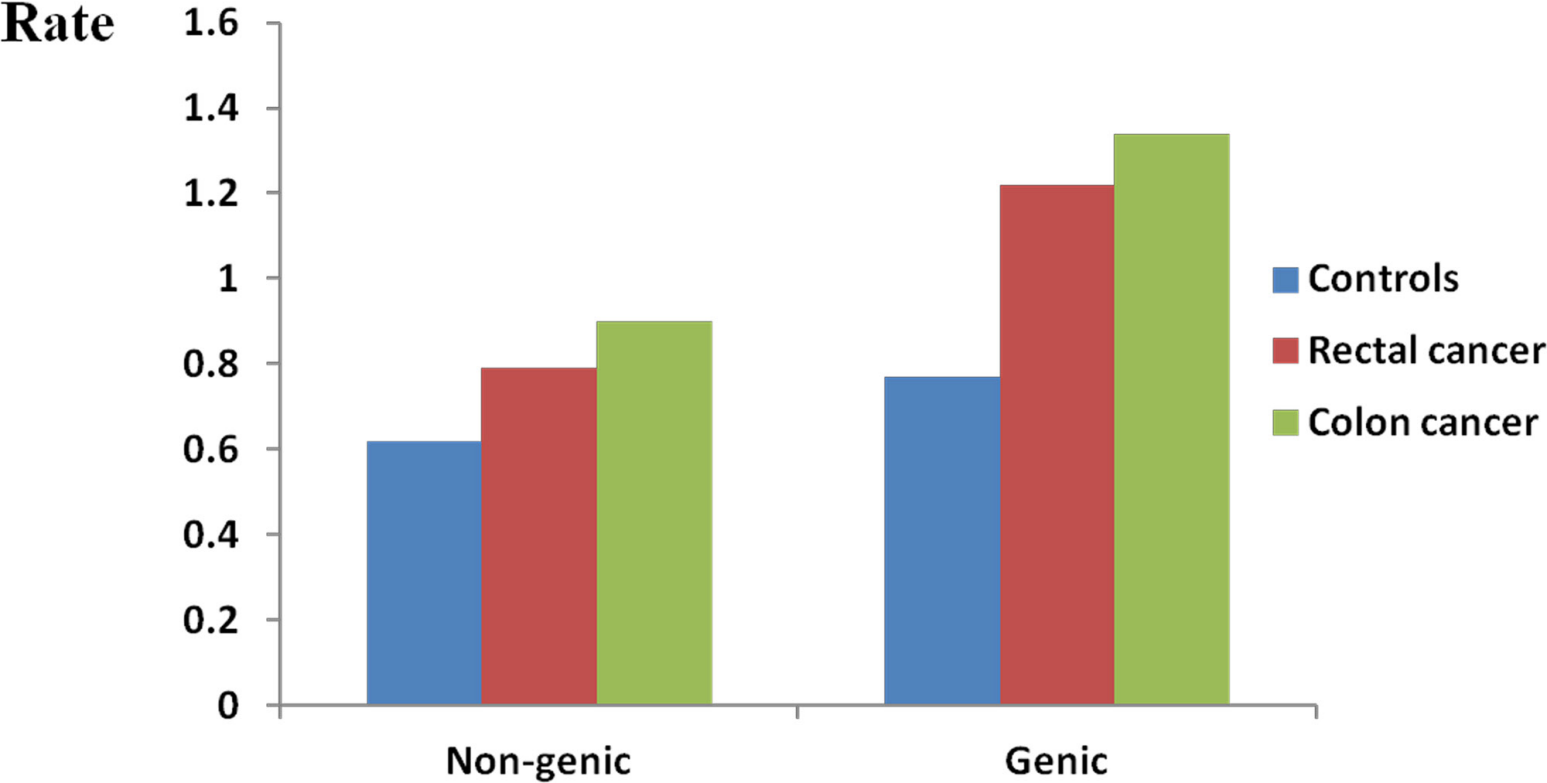
Genome-wide burden of rare non-genic CNVs and genic CNVs Genome-wide frequency of rare genic CNVs and rare non-genic CNVs were calculated for controls, rectal cancer patients and colon cancer patients respectively. Rate (Y axis) represents the number of rare genic/non genic CNVs per individual.

**Figure 3:**
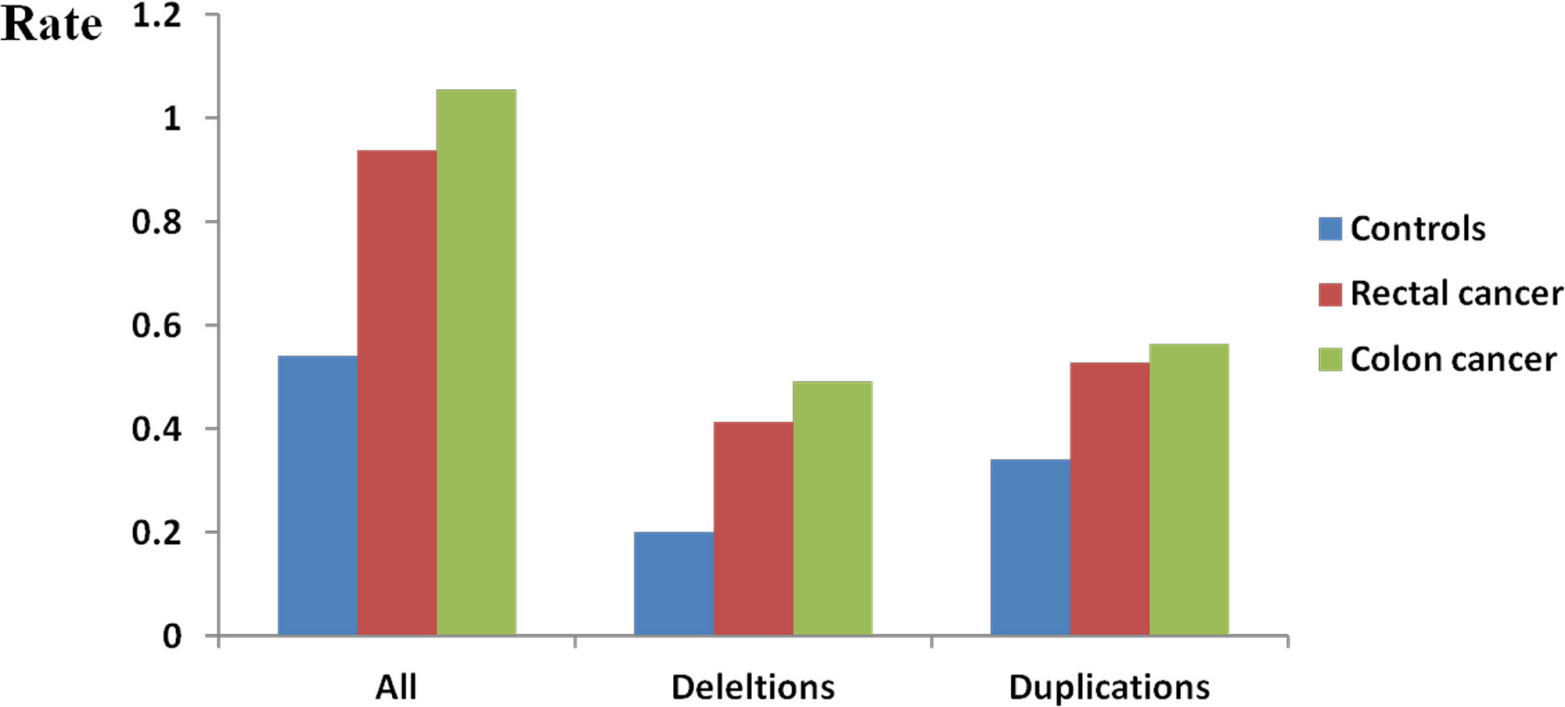
Genome-wide burden of rare CNVs overlapped with coding regions Frequency (Y axis) of all rare CNVs, rare deletions and rare duplications overlapped with coding region were calculated separately. Each cluster consisted of three bars representing controls, rectal cancer patients and colon cancer patients.

In regards to the genic rare CNVs (rare CNVs overlapping with one or more genes as defined in methods), a remarkably higher rate was noted in CRC cases (*P* < 1.0 × 10^−6^, Table 3). Overall, a more pronounced difference in rare genic CNVs compared to global rare CNVs was observed in CRC cases vs. controls (1.65-fold vs 1.53-fold). We observed more apparent frequency difference of genic rare CNVs than non-genic rare CNVs between rectal/colon cancer and controls (Figure 2). Both colon and rectal cancer cases carried more genic rare deletions and duplications than controls did. Interestingly, we detected a significantly higher proportion of colon cancer cases carrying at least one genic deletion, but not duplications (*P* = 8.0 × 10^−6^ and *P* = 0.20, respectively). A significantly higher proportion of both genic deletions and duplications was observed in rectal cancer patients (*P* = 0.005 and *P* = 0.004). Furthermore, we examined the rare CNVs overlapped with protein coding sequences (CDSs). Both the colon cancer patients and the rectal patients carried more such rare deletions/duplications than controls did (Figure 3). An even greater fold change between CRC and controls was observed (1.84-fold, *P* < 1.0 × 10^−6^, Supplementary Table 4).

**Table 3:** Global burden of genic rare CNVs between colorectal cases and controls

Category	Controls (*N* = 1641)	CRC (*N* = 694)	Fold Change^#^	*P* value*	Colon (*N* = 336)	Fold Change^#^	*P* value*	Rectal (*N* = 340)	Fold Change^#^	*P* value*
**Total number of genic CNVs**										
Total	1271	887			451			415		
Deletion	576	449			231			207		
Duplication	695	438			220			208		
**Number of genic CNVs per sample**										
Total	0.77	1.28	1.65	**<1.0 × 10^−6^**	1.34	1.73	**<1.0 × 10^−6^**	1.22	1.58	**<1.0 × 10^−6^**
Deletion	0.35	0.65	1.84	**<1.0 × 10^−6^**	0.69	1.96	**<1.0 × 10^−6^**	0.61	1.73	**<1.0 × 10^−6^**
Duplication	0.42	0.63	1.49	**<1.0 × 10^−6^**	0.65	1.55	**0.00002**	0.61	1.44	**0.00002**
**Proportion of samples with one or more genic CNVs**										
Total	0.52	0.63	1.20	**<1.0 × 10^−6^**	0.63	1.21	**0.0002**	0.63	1.20	**0.0003**
Deletion	0.28	0.38	1.35	**<1.0 × 10^−6^**	0.40	1.45	**8.0 × 10^−6^**	0.35	1.26	**0.005**
Duplication	0.33	0.39	1.15	**0.01**	0.36	1.08	0.20	0.41	1.23	**0.004**
**Total length of genic CNVs spanned per sample (in kb)**										
Total	202.30	206.60	1.02	0.40	178.30	0.88	0.83	236.70	1.17	0.11
Deletion	124.00	119.50	0.96	0.48	121.80	0.98	0.38	114.00	0.92	0.51
Duplication	213.40	220.50	1.03	0.36	175.50	0.82	0.93	263.20	1.23	0.05

* Empirical *p*-values between cases and controls were calculated using 1000,000 permutations by PLINK, and all the *P* values were shown in bold if reached statistical significance (*P* < 0.05).

# Fold change of CRC/colon/rectal cases vs controls.

### Enrichment analysis of the CNV-disrupted genes

We utilized DAVID to examine whether CNV-disrupted genes specific to CRC cases may be enriched in some functional annotations. As a result, 639 genes were disrupted by the CNVs in all CRC cases within our dataset, of which 372 were found in colon cancer patients and 299 in rectal cancer patients. Ultimately, a total of 15 items were significantly enriched after Bonferroni correction (Table 4). The most significant term in the GO analysis was a cellular component (CC) term identified as nucleosome (15 DAVID genes, Bonferroni corrected *P* = 2.80 × 10^−6^). The remaining CC terms were mainly focused on chromatin or DNA. All the biological process (BP) terms were associated with chromatin or DNA assembly, except one term concerned about sensory perception of smell. Olfactory receptor activity, a molecular function (MF) term, was also significantly enriched (31 DAVID genes, Bonferroni corrected *P* = 0.0215).

**Table 4:** Enriched GO functional terms of exclusively disrupted genes in CRC cases

Category ^1^	Term	Count ^2^	% ^3^	*P* Value	Bonferroni
**Rare CNVs exclusive to colorectal cancer**					
GOTERM_CC	GO:0000786~nucleosome	15	2.60	7.95E-09	2.80E-06
GOTERM_CC	GO:0032993~protein-DNA complex	17	2.94	1.05E-08	3.68E-06
GOTERM_BP	GO:0031497~chromatin assembly	16	2.77	6.79E-08	1.30E-04
GOTERM_BP	GO:0006333~chromatin assembly or disassembly	19	3.29	7.81E-08	1.50E-04
GOTERM_BP	GO:0065004~protein-DNA complex assembly	16	2.77	1.26E-07	2.42E-04
GOTERM_BP	GO:0006334~nucleosome assembly	15	2.60	2.89E-07	5.53E-04
GOTERM_BP	GO:0006323~DNA packaging	17	2.94	6.80E-07	1.30E-03
GOTERM_BP	GO:0034728~nucleosome organization	15	2.60	1.04E-06	2.00E-03
GOTERM_CC	GO:0000785~chromatin	22	3.81	1.60E-06	5.64E-04
GOTERM_CC	GO:0005694~chromosome	35	6.06	4.41E-06	1.55E-03
GOTERM_BP	GO:0007608~sensory perception of smell	32	5.54	1.36E-05	2.57E-02
GOTERM_BP	GO:0034622~cellular macromolecular complex assembly	26	4.50	2.17E-05	4.07E-02
GOTERM_MF	GO:0004984~olfactory receptor activity	31	5.36	3.59E-05	2.15E-02
GOTERM_CC	GO:0044427~chromosomal part	29	5.02	4.35E-05	1.52E-02
GOTERM_CC	GO:0045095~keratin filament	12	2.08	1.05E-04	3.64E-02
**Rare CNVs exclusive to colon cancer**					
GOTERM_CC	GO:0032993~protein-DNA complex	16	4.78	6.16E-11	1.69E-08
GOTERM_CC	GO:0000786~nucleosome	14	4.18	1.32E-10	3.62E-08
GOTERM_BP	GO:0006333~chromatin assembly or disassembly	18	5.37	2.66E-10	4.02E-07
GOTERM_BP	GO:0031497~chromatin assembly	15	4.48	8.81E-10	1.33E-06
GOTERM_CC	GO:0000785~chromatin	21	6.27	1.25E-09	3.45E-07
GOTERM_BP	GO:0065004~protein-DNA complex assembly	15	4.48	1.63E-09	2.46E-06
GOTERM_BP	GO:0006323~DNA packaging	16	4.78	5.65E-09	8.54E-06
GOTERM_BP	GO:0006334~nucleosome assembly	14	4.18	5.85E-09	8.83E-06
GOTERM_BP	GO:0034728~nucleosome organization	14	4.18	2.08E-08	3.15E-05
GOTERM_CC	GO:0044427~chromosomal part	25	7.46	3.15E-07	8.67E-05
GOTERM_CC	GO:0005694~chromosome	27	8.06	6.24E-07	1.72E-04
GOTERM_BP	GO:0034622~cellular macromolecular complex assembly	22	6.57	7.35E-07	1.11E-03
GOTERM_CC	GO:0045095~keratin filament	12	3.58	7.91E-07	2.18E-04
GOTERM_BP	GO:0034621~cellular macromolecular complex subunit organization	22	6.57	4.64E-06	6.98E-03
GOTERM_BP	GO:0065003~macromolecular complex assembly	30	8.96	2.56E-05	3.79E-02
GOTERM_CC	GO:0043228~non-membrane-bounded organelle	74	22.09	1.58E-04	4.24E-02
GOTERM_CC	GO:0043232~intracellular non-membrane-bounded organelle	74	22.09	1.58E-04	4.24E-02
**Rare CNVs exclusive to rectal cancer**					
	none				

1 BP, biological process; CC, cellular component; MF, molecular function.

2 Count, number of DAVID gene IDs identified in specific GO terms. Note that the number may be different with the number of Ensembl gene IDs as DAVID incorporates some functionally similar Ensembl gene IDs into one DAVID gene ID according to DAVID Knowledgebase.

3 %, (Count of involved genes / Total number of genes within a particular term) *100.

The 372 and 299 genes disrupted specifically in colon and rectal cancer patients, but not in controls, were further analyzed separately. As a result, 17 terms were overrepresented in colon cancer, mainly focused on the function of nucleosome or chromatin assembly and non-membrane-bounded organelle. However, no significant GO terms survived after Bonferroni correction for rectal cancer.

### Greater rare CNV burden among the younger CRC cases

**Figure 4:**
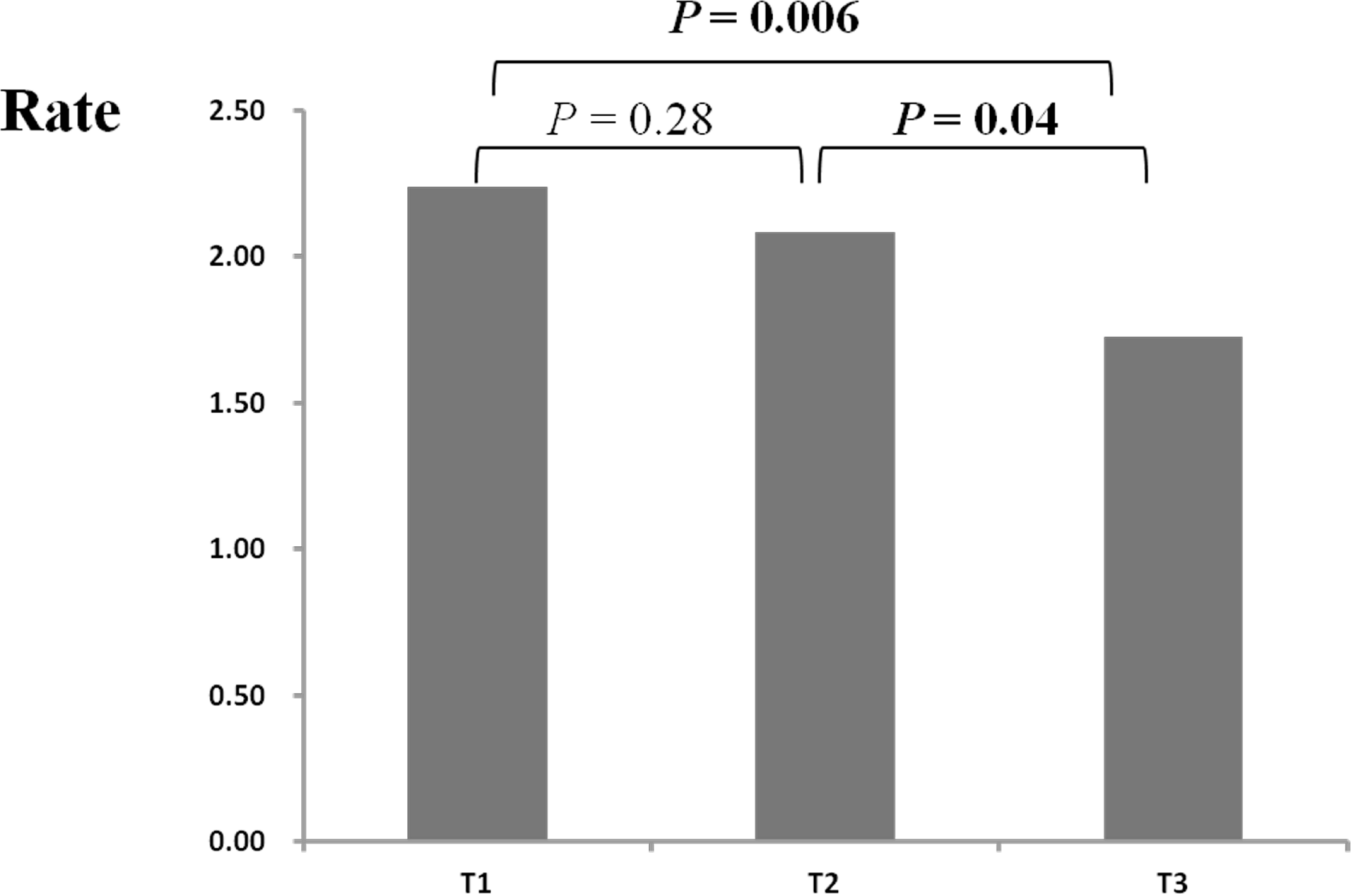
Rate differences of rare CNVs among colorectal cases across different age groups CRC cases were divided into three groups according to case age tertile (T1, T2, T3) (X axis). Rate (Y axis) represents the number of rare CNVs per sample. The *P* values between different age groups were calculated by PLINK.

CRC cases were divided into three groups, according to age tertile within all CRC cases. All the three groups had a higher frequency of rare CNVs than controls did (all *P* < 0.05, data not shown). Both the lowest tertile (age < = 57) and the middle tertile (age between 57 and 69) carried greater burdens of rare CNVs than the highest tertile did (Figure 4, *P* = 0.01 and *P* = 0.06, respectively). However, the frequency of rare CNVs was fairly close among different age groups in control samples (Supplementary Figure 3). Additionally, burden comparison within age groups by decade showed that CRC cases carried a higher burden of rare CNVs than controls within each age subgroup except the oldest group (age > 80) (Supplementary Figure 5). It should be noted that the number of samples aged more than 80 was small (N CRC cases = 42 and N controls = 50). Furthermore, we speculated that the rare CNVs enriched in younger CRC cases may contribute more to CRC. Gene enrichment analysis showed that genes disrupted by rare CNVs were also associated with terms of “nucleosome or chromatin assembly” (Supplementary Table 5). Interestingly, we found that the majority of genes associated with “chromatin assembly or disassembly” were disrupted in younger cases (15 of 17 DAVID genes), indicating the importance of these genes in the pathogenesis of CRC.

### Expression profile analysis

We compared expression difference of 38 genes (number of Ensembl gene IDs) which enriched in the “chromatin assembly or disassembly” item in our study by analyzing published microarray data sets from GEO website. We identified 58 probes corresponding to the 38 genes in both GDS2947 and GDS4382. As a result, approximately 43.1% of the probes were found to be differentially expressed between colorectal adenoma and adjacent normal tissue (GDS2947), and similar results (~41.3%) were observed in another dataset comparing CRC tumors and paired normal tissues (GDS4382) (Supplementary Table 6).

### CNV validation by qPCR

For each CNV, the copy number was determined as the average of 2^−ΔΔCt^ of two pairs of primers. qPCR confirmed all the ten randomly selected rare CNVs, and the results were graphically displayed in Supplementary Figure 4.

## DISCUSSION

To our knowledge, this is the first large scale genome-wide analysis investigating rare CNVs in sporadic CRC, examining 694 sporadic CRC cases and 1641 controls after strict quality control. Results indicated that rare CNVs increased the risk of CRC. Enrichment analysis suggested that the assembly of chromatin or nucleosome-related or olfaction-associated genes specific to CRC cases may contribute to the rising risk of CRC.

The burden analysis revealed remarkably significant associations between rare CNVs and the risk of CRC. When limited in rare genic CNVs, an even greater fold change of overall burden was observed (1.65 vs 1.53). This result would be expected if genic CNVs are a proxy of putatively functional CNVs. One study that investigated CNVs in Parkinson's disease also observed a significantly increased rate of rare genic CNVs in cases compared to controls [26]. Soemdedi *et al* identified an association of rare genic deletions with an increased risk of congenital heart disease [27]. Similar findings were also observed in psychiatric diseases such as autism [28]. Such evidence indicates that rare genic CNVs could be pathogenic in nature and contribute to the pathogenesis of complex disease via affect the expression of the genes. Rare CNVs overlapped with gene coding sequences, which may disrupt protein structure, showed an even greater fold change (1.84-fold vs 1.53). This suggested that rare CNVs overlapped with coding regions could have greater likelihood for causality.

Interestingly, younger cases were inclined to carry a significantly higher burden of rare CNVs than older ones. This finding suggests that rare CNVs may have greater effects on younger affected individuals when compared to older ones. It has been suggested that genetic effects of cancer-related variants differed by age, and younger CRC patients were expected to have a more pronounced genetic predisposition [29, 30].

The genes disrupted exclusively in CRC cases were mainly over-represented in two types of GO terms: assembly of chromatin or nucleosomes and olfactory receptor activity. A recent review summarized that chromosomal instability was an important factor in the development of CRC [31]. The nucleosome is a fundamental unit of the chromatin, consisting of DNA and histones, and nucleosome assembly is crucial for the maintenance of genome stability. Chromatin structure can be regulated by nucleosome assembly, and variations in the factors involved in nucleosome assembly have been implicated in the pathogenesis of human cancer [32]. Frequent mutations of chromatin remodeling pathway were observed in glioblastoma multiforme by Jeremy Schwartzentruber *et al*. Additionally, they found that cases with such mutations carried more CNVs per genome [33]. Importantly, abnormal expression of nucleosome assembly-related genes such as the histone chaperone DEK proto-oncogene (*DEK*), chromatin assembly factor 1 (CAF-1), and chaperone anti-silencing function 1 (Asf1) were involved in the development of cancers [34–36]. Plentiful studies have revealed that DNA copy number change would result in the change of expression level of corresponding genes [37, 38]. The expression profile from GEO data showed that approximately 43.1% and 41.3% of the probes corresponded to “chromatin assembly and disassembly” item were differentially expressed between colorectal adenoma/CRC and adjacent normal tissue. Although rare CNVs may account for a small fraction of variations of gene expression, these results further supported that the DNA assembly-related genes may involve in the development of CRC.

Olfactory receptors (ORs) are G protein-coupled receptors that can detect and discriminate a large variety of aromatic molecules present in the environment. A multigene family mainly expressed in olfactory epithelium that encodes ORs was first discovered by Linda Buck and Richard Axel in 1991 [39]. Later studies revealed that ORs are also expressed in a variety of non-olfactory tissues and have many additional functions, including colonic tissue [40, 41]. OR genes have been associated with several cancers, including breast, prostate cancer and salivary gland carcinoma [42–44]. Of note, a recent study found that ORs could promote the invasiveness and metastasis of cancer cells [45]. Previously, Sturzu A et al. have identified OR1D2 (olfactory receptor, family 1, subfamily D, member 2) as a promising target for prostate cancer [43]. OR4F15 (olfactory receptor, family 4, subfamily F, member 15) have also been found to be associated with salivary gland carcinoma via a GWAS on 309 cases and 535 cancer-free controls [44]. Activation of OR1A2 (olfactory receptor, family 1, subfamily A, member 2) was indicated in hepatocellular carcinoma progression with significant phosphorylation of p38 MAPK and reduced cell proliferation [46]. The aforementioned three OR genes, OR1D2, OR4F15 and OR1A1 were also disrupted in CRC cases but not controls in our study, which underscores the importance of OR activity-associated genes in colorectal cancer.

Seventeen terms mainly focused on nucleosome or chromatin assembly were observed in colon cancer after gene enrichment analysis whereas no significant term was found in rectal cancer. This result was compatible with previous studies in which dysfunction of various signal pathways were varied between colon and rectal cancers, suggesting that the mechanisms of colon and rectal cancer development may not be identical [47, 48]. Burden analysis additionally showed that the colon cancer patients displayed more obvious tendency than rectal cancer patients (Figure 2 and Figure 3). Our results complemented the idea suggested by Burgess RJ et al that colon cancer possessed a stronger genetic component than rectal cancer does [49].

The current study has several limitations. Firstly, no replication in another independent population was conducted, which may result in some bias or chance findings. Although replication of global burden of rare variants is elusive and difficult, further studies involving larger sample size will be of value. Secondly, MS components data for CRC cases was not available. 1641 cancer-free controls consisted of 815 MS controls and 826 non-MS controls. MS is a clustering of metabolic abnormalities with high prevalence of about 30% varying among different populations [50, 51]. Therefore, we think the controls with inclusion of MS controls may better represent the population, although we got similar results when including or excluding MS controls (Table 2 and Supplementary Table 2). Thirdly, small CNVs, which may also have a contribution to CRC, were not evaluated in our study due to the detection limitations of SNP array.

In conclusion, a greater burden of rare CNVs was observed in sporadic CRC cases than controls, and the burden was significantly decreased in older patients. Genes specifically disrupted in colon cancer, but not rectal cancer cases were significantly enriched in DNA assembly and olfactory receptor associated functional categories. These findings suggest that rare CNVs contribute to CRC predisposition and disruption of the OR pathway and DNA assembly play an underlying role in the pathogenesis of CRC.

## MATERIALS AND METHODS

### Study subjects

CRC patients were recruited from The First Affiliated Hospital of Zhejiang University, Sir Run Run Shaw Hospital of Zhejiang University and Taizhou Hospital of Zhejiang Province, who were diagnosed with CRC between 2006–2011. Pathologic diagnoses were evaluated by pathologists via biopsy reports and patients with familial adenomatous polyposis, hereditary non-polyposis CRC and inflammatory bowel disease were excluded. A comprehensive demographic and health survey was carried out among individuals who participated in a large-scale physical examination in the medical center of the Third People's Hospital of Xiaoshan Zhejiang from July 2010 to July 2011. Finally, a total of 1994 cancer-free controls without a family history of cancer (including 998 controls with MS and 996 non-MS controls) were included in our study. The subjects with MS were defined according to the Chinese Diabetes Society (CDS) definition [52]. All participants provided written, informed consent for this study and the ethics committee of Zhejiang University's School of Medicine approved the protocol.

### Genotyping and CNV calling

Genomic DNA was extracted by a TACO automatic nucleic acid extraction apparatus (GeneReach Biotechnology Corp., Taiwan, China). Nano drop 2000 (Thermo Scientific) was used to measure the concentration and purity. Qualified DNA for all samples was genotyped using Illumina Human-Omni Express BeadChip (Illumina Inc., San Diego, CA, USA). To ensure genotyping quality, CRC cases, controls with MS and non-MS controls were mixed in each BeadChip. Forty-six duplicate samples (23 pairs) were genotyped. All the BeadChips were processed in the Bio-X Institute, Shanghai Jiao Tong University. Genotyping procedures were carried out according to the manufacturer's standard protocol.

Different CNV calling algorithms can often produce discrepant results for the same data set. Recent CNV studies have supported a stringent discovery criterion of focusing solely on CNVs that are identified by at least two different programs [53–55]. Therefore, CNV segments were identified by both Penncnv and Quantisnp in our study [56, 57]. Both of the two algorithms are based on a Hidden Markov Model (HMM), using intensity files generated by GenomeStudio software from Illumina. QuantiSNP2.0, based on an objective Bayes HMM and takes into consideration log R Ratio (LRR) as well as B allele frequency (BAF) of each SNP. PennCNV algorithm incorporates additional information including population frequency of B allele (PFB) and the distance between adjacent SNPs. To reduce false positive calls due to genomic waves, GC-content adjustment was performed to correct the bias in both analysis [58]. Default settings for both algorithms were applied. In addition, adjacent CNV segments with same copy number were merged into a single call if the length of gap in between was shorter than half of total length of the two consecutive CNVs.

### Sample quality control

To provide reliable results, samples with genotype rates of less than 95% or outliers were removed. Further criteria for the exclusion of noisy data were applied respectively for each algorithm. Samples were further excluded when meet one of the following criteria: individuals with more than 200 CNVs; an absolute value of GC wave factor (GCWF) larger than 0.05 or an standard deviation of LRR > 0.3, as recommended by PennCNV; a genome-wide LRR SD obtained from QuantiSNP greater than 3.5. Principle component analysis (PCA) was performed by Eigenstrat to examine ancestry in our study and outliers were excluded [59].

### CNV quality control

To obtain high-confidence calls CNVs, we only remained CNVs satisfied all the following criteria: with a maximum Bayes factor >10 predicted by Quantisnp; possessing identical breakpoints identified by both Quantisnp and PennCNV; CNVs of larger than 10 kb and spanning ten or more contiguous probes.

### Burden analysis

Considering our moderate sample size, rare CNVs were defined as those with a frequency of <0.5% in our dataset [23]. In order to evaluate the overall differences of CNV distribution between cases and controls, CNV burden analyses were conducted by PLINK [60], using 100,0000 permutations.

### Functional annotation of CRC-specific CNVs

Functional annotation was explored for genes specifically disrupted by CNVs in CRC patients (CRC-specific) by an online Database for Annotation, Visualization and Integrated Discovery (DAVID) [61]. Genes were determined by RefSeq annotations (UCSC, v. July 2008, NCBI v36, hg18) and gene boundaries were extended with a 10 kb flanking region on either side as referred by Pinto D et al [28]. The gene ontology (GO) functional annotation was run with a default setting and the functional items with *P* value < 0.05 after Bonferroni correction were presented in the results.

### Expression profile analysis

Datasets with gene expression profile comparing CRC or colorectal adenoma to paired adjacent normal tissue were obtained from Gene Expression Omnibus (GEO) database. Dataset GDS4382 was utilized to compare 17 paired CRC and adjacent normal tissue samples [62]. And the comparison between 32 paired colorectal adenoma and adjacent normal tissue samples were performed by dataset GDS2947 [63]. Both the two datasets were based on the Affymetrix Human GenomeU133 Plus 2.0 Array. The expression data of the probes corresponding to genes in significant GO terms from functional annotation analysis were extracted, and Wilcoxon paired test was performed for each probe.

### CNV validation by qPCR

Quantitative real-time PCR (qPCR) was performed to measure the copy number of rare CNVs. RNase P was used as an endogenous reference. Ten rare CNVs were randomly selected, and two pairs of primers were designed for each CNV segment (primers are shown in Supplementary Table 7). Five samples were examined for each CNV (one with a putative deletion/duplication, the remaining four with two putative copies). qPCR was performed in triplicates on a LightCycler^®^ 480 Instrument (Roche, Mannheim, Germany) using SYBR-green dye. Finally, the “delta delta Ct” method was used to calculate the relative copy numbers at target regions [64].

## SUPPLEMENTARY FIGURES AND TABLES


