# Supplementary material for: A genome-wide assessment of rare copy number variants in colorectal cancer
Source: Oncotarget. 2015 Jul 20;6(28):26411–23. doi: 10.18632/oncotarget.4621 (PMC4694911; doi:10.18632/oncotarget.4621)
Supplement: Supplementary file 1 [file oncotarget-06-26411-s001.pdf]

SUPPLEMENTARY FIGURES AND TABLES

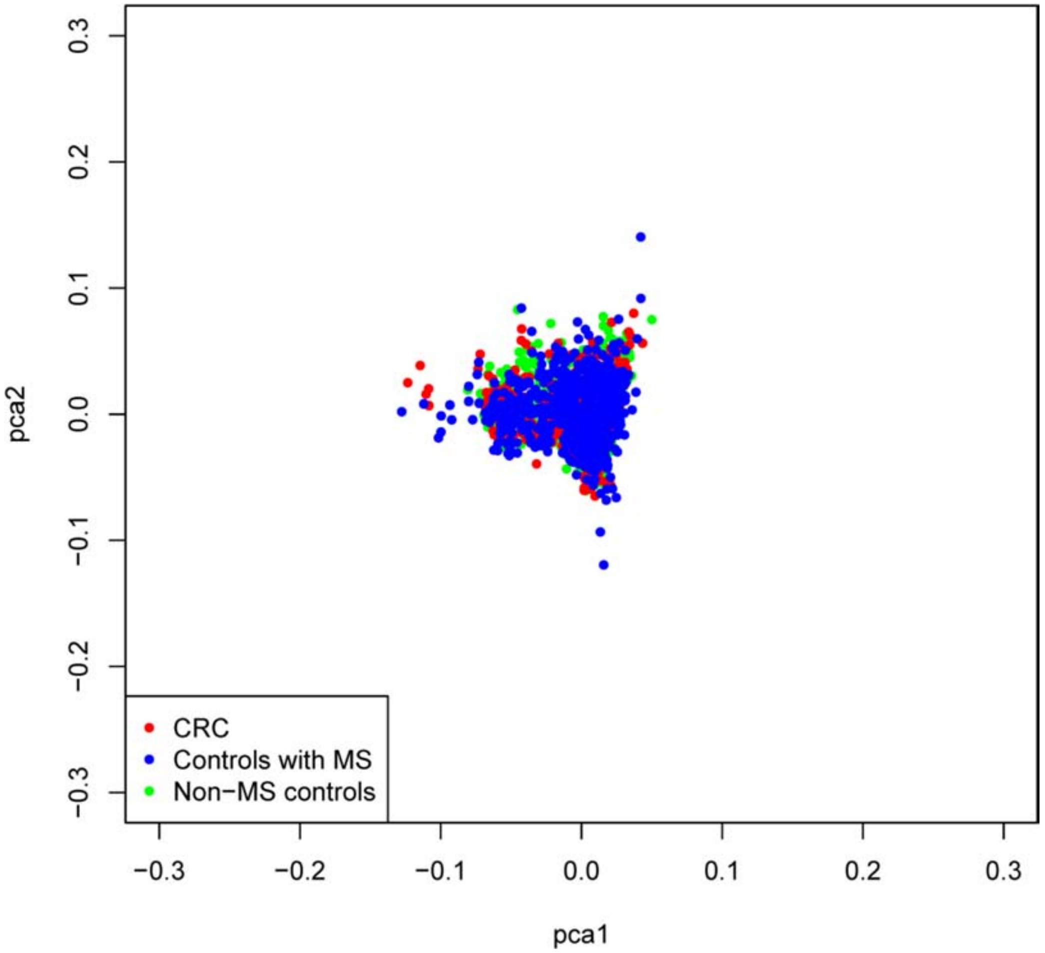

Supplementary Figure S1: Principle Components Analysis of cases and controls. The first two principal components of CRC cases, MS controls and non-MS controls obtained by PCA analysis were plotted. Each dot represented one of the 2335 individuals and different colored dots were used for representing different types of samples. None of the samples was removed as an outlier according to the principal components analysis.

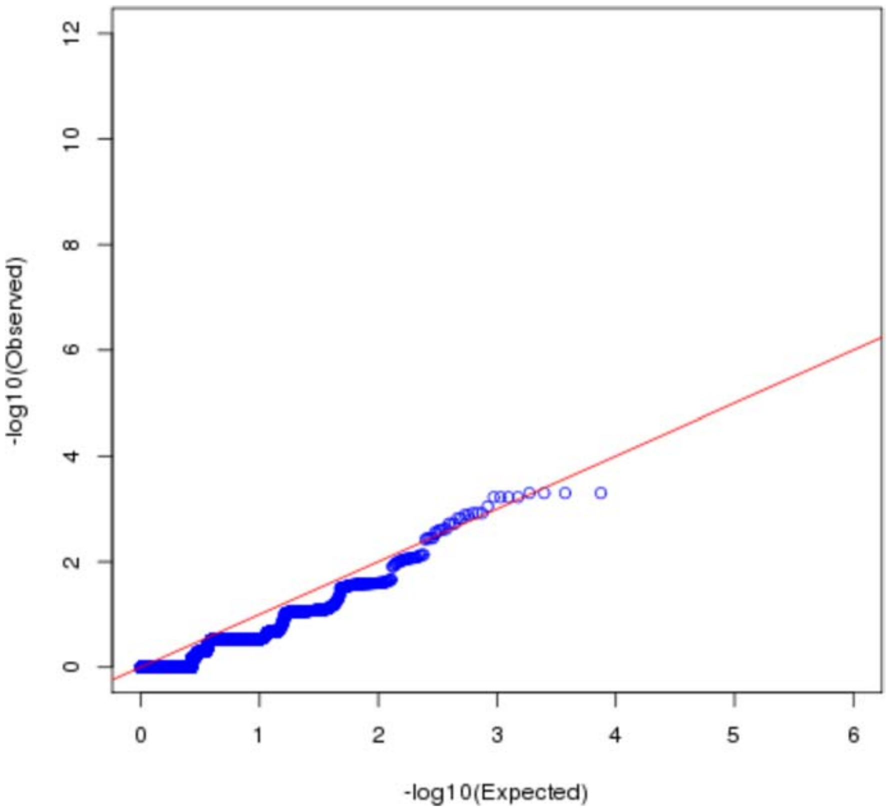

Supplementary Figure S2: QQ-plot of rare CNVs spanning a particular position. The results were based on the empirical p-values calculated for each position (at least one rare CNV in the position) by PLINK, using 100,0000 permutations.

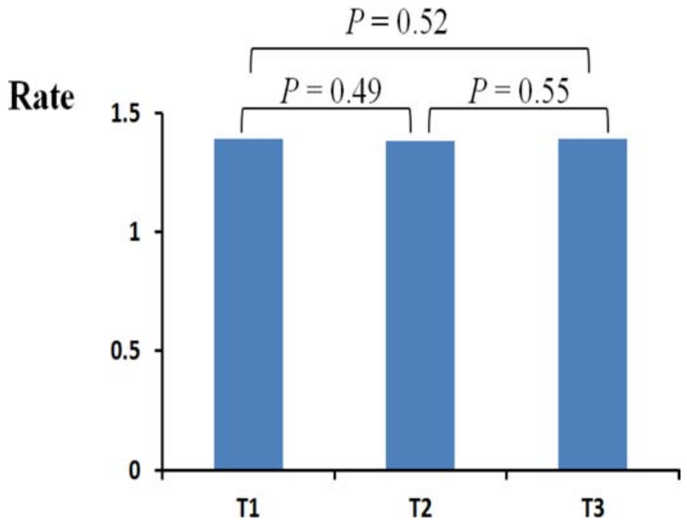

Supplementary Figure S3: Rate difference of rare CNVs between controls across different age groups. All control samples after quality control were divided into three groups according to age tertile (T1, T2, T3) (*X* axis). Rate (*Y* axis) represents the number of rare CNVs per sample. The *P* values between different age groups were calculated by PLINK. The frequency of rare CNVs was fairly close among different age groups.

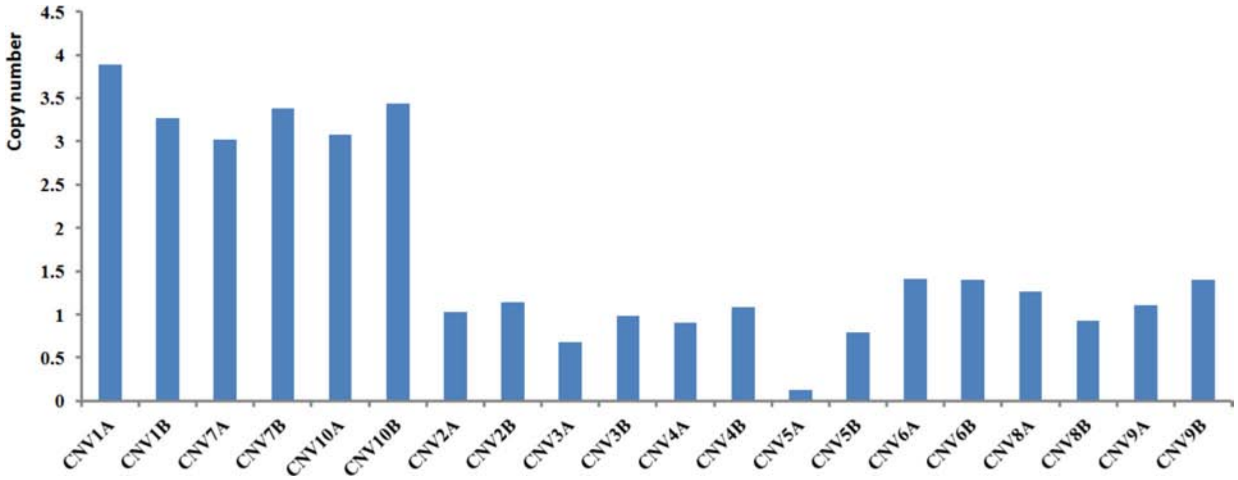

Supplementary Figure S4: Validation of ten randomly selected rare CNVs by qPCR. Two pairs of primers were designed for each CNV to improve the accuracy. Five samples were examined for each CNV (one with putative deletion/duplication, the remaining four with two copies).The copy number (*Y* axis) for each CNV was calculated as the average of the relative copy number determined by the two pairs of primers. These results were all consistent with the CNVs called by PennCNV and QuantiSNP.

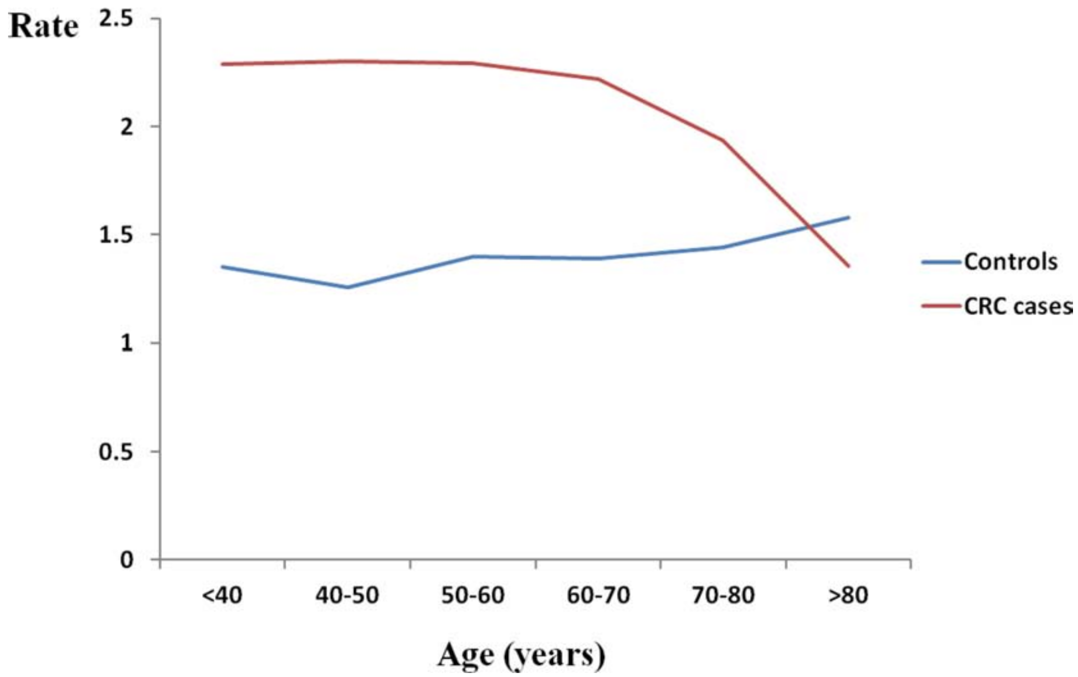

Supplementary Figure S5: Comparison of CNV burden between CRC cases and controls according to age by decade. Rate (*Y* axis) represents the number of rare CNVs per individual in each age group (*X* axis).

Supplementary Table S1. Primer sequences for rare CNVs and reference gene

ID	Region*	Forward Primers (5'-3')	Reverse Primers (5'-3')	Type
CNV1A	chr1:45898467-46198087	TTGAGGCAGATTTCAGTGGG	TGGCTGTAGTTGCTGTTGTC	Dup
CNV1B	chr1:45898467-46198087	GCATCTTGGGTCTCATTC	TTCTAAGCGTTGGTCTCC	Dup
CNV2A	chr11:120212854-120301098	TACCAAAGTCCATCCCTC	CCACAAGGCTGTCCATTC	Del
CNV2B	chr11:120212854-120301098	CATTGACGATGGGCTCTG	CCTCTTCCCTTTCTTACC	Del
CNV3A	chr19:51466738-51550401	GAACAGAACAGGACGGAATG	TTTGGATGGAGGGTTGGAGC	Del
CNV3B	chr19:51466738-51550401	CTATGTGCCTCCCTTACC	AGAGCGAGCAGTGCCTTC	Del
CNV4A	chr14:82569220-82657050	CCCAGGAAAGGGTGAATG	GTGCCTCACGGTTGCTTC	Del
CNV4B	chr14:82569220-82657050	TGTTGCCCTTCTGCCTTTC	GGAGGCTGGGAGCATTTTC	Del
CNV5A	chr2:56605078-56719522	ATTGATGGTCCCTGTGAG	GCACCTAAGGCAGTAAGC	Del
CNV5B	chr2:56605078-56719522	CTCCTTCCCTACATCGAC	TATCCAGACAGCAGCAAC	Del
CNV6A	chr2:124766475-12488029	GCTCACCGTTATTCCTAC	CTGGTCCTGTTGACATCG	Del
CNV6B	chr2:124766475-12488029	TTCCACAGCCCTCCTATC	CCCTCACTCCTGATTCTC	Del
CNV7A	chr3:164820983-165217908	TGTTTAGCCAGAAGTCGC	CAAAGCAGGGATAGAAGG	Dup
CNV7B	chr3:164820983-165217908	AGAAAGCAGACAGGGAGG	ACCACAGTTGGCAAGAGG	Dup
CNV8A	chr3:11329796-113329041	ATCCCATCCATGACCACC	AGAGCCCTTAAACGCCAG	Del
CNV8B	chr3:11329796-113329041	CTGTGCATGGGTTAGGAG	TGTCTATGAGGCTTGGTG	Del
CNV9A	chr4:27369239-27491240	GCAATAAGTAGCCAGGAC	AGCCAGCAACGTGGTAAG	Del
CNV9B	chr4:27369239-27491240	TTTGCTGGGAAGTAGTGC	GAAAAGAGGGCGTAGAAG	Del
CNV10A	chr13:26813705-26887415	GACCAGACCAAGCCATCC	GGCAACAGCCCACATTCC	Dup
CNV10B	chr13:26813705-26887415	GCCCTGTGACCCTGGTTG	AATGCTTGCCTGGCTGTG	Dup
Refernce	RNase P	TATTCACAAAGAGCCCAGAG	GAAGGGTATGGGAAAACAAG	Refernce

*Chromosome positions were based on 2006 (NCBI36/hg18).

Supplementary Table S2. Basic characteristics of MS controls and non-MS controls

	MS controls	Non-MS controls	*P* value[a]
N	815	826	
Gender(M/F)	422/393	428/398	0.99
Age(years)	59.8 ± 10.7	55.0 ± 12.0	<0.001

[a]The *P* value for gender was calculated by χ^2 test between all the cases and controls, while the *P* value of the age between the two groups was derived from independent T test.

Supplementary Table S3. Global burden of rare CNVs between colorectal cases and non-MS controls

Category	Controls ($N = 826$)	CRC ($N = 694$)	Fold Change#	*P* value*	Colon ($N = 336$)	Fold Change#	*P* value*	Rectal ($N = 340$)	Fold Change#	*P* value*
Total number of rare CNVs										
Total	1121	1394			721			641		
Deletion	593	810			417			371		
Duplication	528	584			304			270		
Number of rare CNVs per sample										
Total	1.36	2.01	1.48	**$<1.0 \times 10^{-6}$**	2.15	1.58	**$<1.0 \times 10^{-6}$**	1.89	1.39	**$<1.0 \times 10^{-6}$**
Deletion	0.72	1.17	1.63	**$<1.0 \times 10^{-6}$**	1.24	1.73	**$<1.0 \times 10^{-6}$**	1.09	1.52	**0.00001**
Duplication	0.64	0.84	1.32	**0.0003**	0.90	1.42	**0.0005**	0.79	1.24	**0.005**
Proportion of samples with one or more rare CNVs										
Total	0.74	0.79	1.06	**0.02**	0.79	1.07	**0.04**	0.78	1.06	0.07
Deletion	0.51	0.57	1.11	**0.02**	0.58	1.14	**0.02**	0.55	1.08	0.11
Duplication	0.46	0.49	1.06	0.16	0.46	1.00	0.53	0.52	1.12	**0.05**
Total length of rare CNVs spanned per sample (in kb)										
Total	258.40	256.30	0.99	0.53	253.00	0.98	0.56	260.50	1.01	0.46
Deletion	161.40	156.60	0.97	0.57	173.90	1.08	0.32	137.10	0.85	0.76
Duplication	232.80	228.20	0.98	0.58	211.40	0.91	0.79	245.90	1.06	0.32

*Empirical *p*-values between cases and controls were calculated using 1000,000 permutations by PLINK, and all the *P* values were shown in bold if reached statistical significance ($P < 0.05$).
#Fold change of CRC/colon/rectal cases vs controls.

Supplementary Table S4. Frequency differences of rare CNVs between males and females

Samples	Males	Females	*P* value*
All	1.638	1.574	0.184
CRC	2.247	2.016	0.105
Controls	1.398	1.375	0.363

*Empirical *p*-values for frequency difference between females and males were calculated using 1000,000 permutations by PLINK.

Supplementary Table S5. Global burden of rare CNVs overlapping CDS

Category	Controls (N = 826)	CRC (N = 694)	Fold Change[#]	P value[*]	Colon (N = 336)	Fold Change[#]	P value[*]	Rectal (N = 340)	Fold Change[#]	P value[*]
Total number of rare CNVs										
Total	886	688			354			319		
Deletion	328	312			165			140		
Duplication	558	376			189			179		
Number of rare CNVs per sample										
Total	0.54	0.99	1.84	**<1.0 × 10−6**	1.05	1.95	**<1.0 × 10−6**	0.94	1.74	**<1.0 × 10−6**
Deletion	0.20	0.45	2.25	**<1.0 × 10−6**	0.49	2.46	**<1.0 × 10−6**	0.41	2.06	**<1.0 × 10−6**
Duplication	0.34	0.54	1.59	**<1.0 × 10−6**	0.56	1.65	**9.0 × 10−6**	0.53	1.55	**3.0 × 10−6**
Proportion of samples with one or more rare CNVs										
Total	0.40	0.53	1.31	**<1.0 × 10−6**	0.51	1.26	**0.0003**	0.55	1.37	**3.0 × 10−6**
Deletion	0.17	0.27	1.57	**2.0 × 10−6**	0.28	1.64	**0.00001**	0.26	1.52	**0.0002**
Duplication	0.28	0.34	1.21	**0.003**	0.32	1.13	0.11	0.36	1.29	**0.002**
Total length of rare CNVs spanned per sample (in kb)										
Total	215.60	206.30	0.96	0.64	183.50	0.85	0.86	228.80	0.31	1.06
Deletion	145.30	125.20	0.86	0.66	135.30	0.93	0.44	111.90	0.74	0.77
Duplication	221.90	223.30	1.01	0.47	175.50	0.79	0.94	268.70	0.08	1.21

[*]Empirical p-values between cases and controls were calculated using 1000,000 permutations by PLINK, and all the P values were shown in bold if reached statistical significance ($P < 0.05$).
[#]Fold change of CRC/colon/rectal cases vs controls.

Supplementary Table S6. Enriched GO functional terms of disrupted genes in younger CRC cases

Category[1]	Term	Count[2]	%[3]	*P* Value	Bonferroni
GOTERM_CC	GO:0032993~protein-DNA complex	16	3.57	2.32E-09	7.44E-07
GOTERM_CC	GO:0000786~nucleosome	14	3.13	3.22E-09	1.03E-06
GOTERM_CC	GO:0005694~chromosome	33	7.37	1.07E-07	3.45E-05
GOTERM_BP	GO:0006333~chromatin assembly or disassembly	17	3.79	5.98E-08	9.83E-05
GOTERM_BP	GO:0006334~nucleosome assembly	14	3.13	9.10E-08	1.50E-04
GOTERM_CC	GO:0000785~chromatin	20	4.46	4.84E-07	1.55E-04
GOTERM_BP	GO:0031497~chromatin assembly	14	3.13	1.40E-07	2.29E-04
GOTERM_BP	GO:0065004~protein-DNA complex assembly	14	3.13	2.40E-07	3.94E-04
GOTERM_BP	GO:0034728~nucleosome organization	14	3.13	3.11E-07	5.11E-04
GOTERM_CC	GO:0044427~chromosomal part	27	6.03	3.24E-06	1.04E-03
GOTERM_BP	GO:0006323~DNA packaging	15	3.35	7.69E-07	1.26E-03
GOTERM_CC	GO:0045095~keratin filament	12	2.68	9.80E-06	3.14E-03

[1]BP, biological process; CC, cellular component; MF, molecular function.
[2]Count, number of DAVID gene IDs identified in specific GO terms. Note that the number may be different with the number of Ensembl gene IDs as DAVID incorporates some functionally similar Ensembl gene IDs into one DAVID gene ID according to DAVID Knowledgebase.
[3]%, (Count of involved genes / Total number of genes within a particular term)*100.

Supplementary Table S7. Expression differences of DNA assembly-related genes in GDS2947 and GDS4382

Probe ID	Gene symbol	*P* [a]	*P* [b]
223556_at	*HELLS*	7.95E-07	2.93E-04
236278_at	*HIST1H3E*	7.95E-07	1.68E-02
206110_at	*HIST1H3H*	5.05E-06	4.18E-03
227350_at	*HELLS*	1.02E-05	1.91E-02
220085_at	*HELLS*	4.96E-05	7.13E-04
244443_at	*CHD2*	1.09E-04	6.87E-01
214616_at	*HIST1H3E*	4.39E-04	3.09E-03
208527_x_at	*HIST1H2BE*	1.91E-03	7.95E-01
205062_x_at	*ARID4A*	1.91E-03	2.77E-02
214481_at	*HIST1H2AM*	2.30E-03	5.54E-01
230141_at	*ARID4A*	7.09E-03	1.49E-01
214472_at	*HIST1H3D,HIST1H2AD*	1.22E-02	6.19E-01
227349_at	*HELLS*	1.76E-02	5.03E-04
230156_x_at	*CHD2*	2.25E-02	1.36E-01
214537_at	*HIST1H1D*	3.30E-02	5.62E-03
208515_at	*HIST1H2BM*	4.34E-02	3.32E-01
210387_at	*HIST1H2BG*	4.75E-02	1.13E-01
208576_s_at	*HIST1H3B*	4.75E-02	2.10E-01
214469_at	*HIST1H2AE*	5.41E-02	9.43E-01
214463_x_at	*HIST1H4J*	6.15E-02	9.29E-02
225077_at	*CHD2*	7.57E-02	3.81E-01
214522_x_at	*HIST1H3D,HIST1H2AD*	7.88E-02	1.13E-01
214534_at	*HIST1H1B*	7.88E-02	1.68E-02
239041_at	*HIST1H2AK*	7.88E-02	1.91E-02
203461_at	*CHD2*	9.99E-02	1.93E-01
203098_at	*CDYL*	1.30E-01	1.02E-01
214562_at	*HIST1H4L*	1.72E-01	8.68E-01
208569_at	*HIST1H2AB*	1.84E-01	8.31E-01
215779_s_at	*HIST1H2BG*	1.91E-01	1.93E-01
207611_at	*HIST1H2BL*	1.91E-01	9.06E-01
203100_s_at	*CDYL*	2.10E-01	3.95E-02
214516_at	*HIST1H4B*	2.10E-01	5.99E-04
214644_at	*HIST1H2AK*	2.17E-01	6.15E-02
206951_at	*HIST1H4E*	2.24E-01	5.62E-03
208547_at	*HIST1H2BB*	2.46E-01	3.56E-01

(*Continued*)

Probe ID	Gene symbol	*P* [a]	*P* [b]
214542_x_at	*HIST1H2AI*	2.62E-01	7.23E-01
208484_at	*HIST1H1A*	2.78E-01	8.40E-02
208580_x_at	*HIST1H4J,HIST1H4K*	2.86E-01	3.52E-02
226830_x_at	*CHD2*	2.86E-01	3.60E-03
214646_at	*HIST1H3J*	3.60E-01	1.49E-01
1554014_at	*CHD2*	3.69E-01	3.81E-01
211484_s_at	*DSCAM*	4.11E-01	1.77E-01
226366_at	*SHPRH*	4.32E-01	8.46E-04
208046_at	*HIST1H4A*	4.54E-01	6.19E-01
208583_x_at	*HIST1H2AJ*	5.75E-01	9.81E-01
228999_at	*CHD2*	5.88E-01	3.32E-01
214554_at	*HIST1H2AL*	5.88E-01	1.63E-01
208490_x_at	*HIST1H2BF*	6.54E-01	8.68E-01
234040_at	*HELLS*	6.54E-01	5.52E-02
214540_at	*HIST1H2BO*	6.95E-01	4.95E-02
237268_at	*DSCAM*	6.95E-01	3.81E-01
208575_at	*HIST1H3A*	7.22E-01	9.81E-01
208076_at	*HIST1H4D*	8.22E-01	1.77E-01
214509_at	*HIST1H3I*	8.37E-01	6.53E-01
1554015_a_at	*CHD2*	8.52E-01	6.19E-01
240218_at	*DSCAM*	8.52E-01	1.00E-03
207226_at	*HIST1H2BN*	8.66E-01	5.52E-02
203099_s_at	*CDYL*	1.00E + 00	2.17E-02

[a, b]*P* values were calculated by Wilcoxon matched-pairs signed-ranks test comparing the expression value of each probe between colorectal adenoma/colorectal cancer with paired adjacent normal tissue in GDS 2947 and GDS 4382 respectively.

Supplementary Table S8. Global burden of rare CNVs between MS controls and non-MS controls

Category	MS controls (N=815)	Non-MS contols (N=826)	Fold Change #	*P value* *	
Total number of rare CNVs					
	Total	1156	1163		
	Deletion	589	610		
	Duplication	567	553		
Number of rare CNVs per sample					
	Total	1.42	1.41	0.99	0.44
	Deletion	0.72	0.74	1.02	0.63
	Duplication	0.70	0.67	0.96	0.27
Proportion of samples with one or more rare CNVs					
	Total	0.74	0.74	1.00	0.57
	Deletion	0.48	0.51	1.07	0.90
	Duplication	0.48	0.48	0.99	0.47
Total length of rare CNVs spanned per sample (in kb)					
	Total	213.4	259.4	1.22	0.97
	Deletion	118.2	161.1	1.36	0.99
	Duplication	211	231.1	1.10	0.84

* Empirical p-values between cases and controls were calculated using 1000,000 permutations by PLINK, and all the *P* values were shown in bold if reached statistical significance ($P < 0.05$).
# Fold change of CRC/colon/rectal cases vs controls.
